# Epstein-Barr virus miR-BART1-3p suppresses apoptosis and promotes migration of gastric carcinoma cells by targeting DAB2

**DOI:** 10.7150/ijbs.36595

**Published:** 2020-01-14

**Authors:** Kyoungmi Min, Jun Yeob Kim, Suk Kyeong Lee

**Affiliations:** Department of Medical Life Sciences, Department of Biomedicine & Health Sciences, College of Medicine, The Catholic University of Korea, Seoul, Republic of Korea

**Keywords:** Epstein-Barr virus, BART miRNA, DAB2, cell proliferation, cell migration

## Abstract

Although Epstein-Barr virus (EBV) is known to encode over 40 different miRNAs of its own, the roles of most EBV miRNAs remain unknown. Disabled homolog 2 (DAB2) is a putative tumor suppressor, but its role in gastric carcinoma (GC), especially in EBV-associated GC, needs to be clarified. Our qRT-PCR and mRNA microarray results showed that DAB2 expression was down-regulated in EBV-positive GC cells compared to EBV-negative cells. Four BART miRNAs that might target DAB2 were predicted, and we found, using a luciferase reporter assay, that miR-BART1-3p directly targeted the 3'-UTR of DAB2. The miR-BART1-3p transfection decreased DAB2 expression at both mRNA and protein levels, while transfection of an inhibitor of miR-BART1-3p, miR-BART1-3p(i), increased DAB2 expression. In addition, miR-BART1-3p as well as siDAB2 increased migration and decreased apoptosis. Meanwhile, miR-BART1-3p(i) or pcDNA3.1-DAB2 transfection decreased migration and increased apoptosis in EBV-infected GC cells. Furthermore, decreased migration by miR-BART1-3p(i) was abrogated by co-transfected siDAB2, while decreased migration by miR-BART1-3p(i) was further suppressed by a co-transfected DAB2 over-expression vector. Our data suggest that miR-BART1-3p plays an important role in the tumorigenesis of EBV-associated GC by directly targeting DAB2.

## Introduction

Gastric carcinoma (GC) is the third most common cause of death (8.2% of all cancer deaths), and the fifth most frequently diagnosed cancer 1. To improve diagnosis or therapeutic intervention, GC has been classified in several different ways 2, 3. With the development of genomic technology and high-throughput sequencing, a new classification system for GC was proposed recently 4. GC cases are divided into four subgroups based on their genomic and molecular characteristics: Epstein-Barr virus (EBV)-infected, microsatellite instable (MSI), genomically stable (GS), and chromosomal instable (CIN) 4. EBV-associated GC (EBVaGC) comprises about 9 to 10% of all GC cases, and this proportion is not significantly different worldwide 5.

More than 90% of human populations around the world are known to be infected with EBV. EBV establishes lifelong infection in the host, indicating that EBV may counteract the effects of host immune surveillance 6, 7. Multiple studies have demonstrated that EBV is closely associated with several tumors, including GC, Burkitt's lymphoma, nasopharyngeal carcinoma (NPC), and Hodgkin's disease. Following infection, the EBV genome is maintained extrachromosomally as an episome, and replicates using cellular replication machinery 8-10. EBVaGC expresses a restricted number of EBV latent genes, including EBV-encoded small RNA (EBER)-1 and -2, EBV nuclear antigen (EBNA)-1, latent membrane protein-2A (LMP2A), and BART miRNAs 11. The expression of EBNA-1 decreased p53 activation by the loss of promyelocytic leukemia nuclear bodies, resulting in an impaired response to DNA damage and consequently promoting cell survival in EBVaGC cells 12. LMP2A increased survivin by activating the nuclear factor-κB (NF-κB) pathway for resistance from serum deprivation-induced apoptosis in GC cells 13. At least 44 mature BART miRNAs have been identified 14. BART miRNAs are expressed at high levels in all EBV-associated carcinomas such as NPC and GC. Accumulating evidence has revealed that several BART miRNAs play important roles in carcinoma development 14, 15. However, the roles of most BART miRNAs in tumorigenesis still require further investigation.

Disabled homolog 2 (DAB2) encodes a mitogen-responsive phosphoprotein that contains a phosphotyrosine-binding domain and a proline-rich domain involved in the regulation of clathrin-mediated endocytosis as well as receptor-mediated signaling pathways 16-20. DAB2 has been considered as a putative tumor suppressor in NPC 21, 22 as well as ovarian 23, 24, breast 25-27, prostate 28, and lung carcinoma 29, 30. Several human miRNAs have been reported to target DAB2, causing tumor promotion. Up-regulated miR-187 suppressed DAB2 and promoted cell proliferation 24. The miR-93-directed down-regulation of DAB2 was shown to be the most important element that contributed to lung cancer tumorigenesis 30. In addition, miR-93 promoted cell growth and invasion in NPC 22. High expression of miR-106b was found in cervical cancer, and miR-106b promoted TGF-β1-induced cervical cancer cell migration by targeting DAB2 31. Further, miR-191 promoted the cellular viability of estrogen receptor-positive breast cancer cells by directly suppressing the expression of DAB2 25. Additionally, the induction of DAB2 expression reduced cancer growth, migration, and invasion 21-25, 27-31.

However, there are few published studies showing DAB2 expression and function in EBVaGC. In this study, we observed reduced DAB2 expression in EBV-positive GC cells and investigated the EBV miRNAs involved in DAB2 down-regulation to clarify the roles of EBV BART miRNAs in EBVaGC.

## Materials and Methods

### Cell cultures and reagents

MKN-1, MKN-28, NCI-N87, SNU-216, and AGS are EBV-negative GC cell lines, while SNU-719, YCCEL1, and AGS-EBV are EBV-positive GC lines. The GC cell lines were cultured in RPMI-1640 containing 10% fetal bovine serum, 100 U/ml penicillin, and 100 μg/ml streptomycin except for YCCEL1, which was cultured in Eagle's minimal essential medium. AGS-EBV is a GC cell line derived from AGS cells by infecting them with a recombinant Akata virus. To culture AGS-EBV cells, 400 μg/ml of G418 (Gibco, Carlsbad, CA, USA) was added to the medium. The human embryonic kidney cell line HEK293T was cultured in Dulbecco's modified Eagle's medium (DMEM) supplemented with 10% FBS, 100 U/ml penicillin, and 100 μg/ml streptomycin. All cells were incubated at 37 °C and supplemented with 5% CO_2_.

### Microarray analysis

Microarray analysis results obtained previously 32 were used for analysis. The relative expressions of tumor suppressor genes including DAB2 were assessed in AGS-EBV cells by comparing the values with those in AGS cells.

### Target prediction

The DAB2 sequence used for miRNA target prediction was extracted from the National Center for Biotechnology Information (NM_001244871.1; Bethesda, MD, USA). To determine whether the 3′-UTR of DAB2 can be targeted by EBV BART miRNAs, we used a publicly available RNA hybrid program (http://bibiserv.techfak.uni-bielefeld.de/rnahybrid/), which is a tool for finding the minimum free energy hybridization of miRNAs to target RNAs**.**

### Transfection of miRNA mimics and miRNA inhibitors

All of the BART miRNA mimics and the scrambled control were purchased from Genolution Pharmaceuticals (Seoul, South Korea). The locked nucleic acid (LNA) inhibitor against miR-BART1-3p and the negative-control LNA-miRNA inhibitor were purchased from Exiqon (Vedbaek, Denmark). The mirVana^TM^ miRNA inhibitor for miR-BART1-3p and the control inhibitor were purchased from Invitrogen (Carlsbad, CA, USA). Two inhibitors showed comparable effects under the conditions used in our experiments. All transfection experiments were performed using Lipofectamine 2000 (Invitrogen) according to the manufacturer's protocol. Protein and RNA were extracted 48 h after transfection.

### Plasmid construction

The full-length 3'-UTR of DAB2 was amplified from the cDNA of AGS-EBV cells. The amplicon was then cloned into the XhoI/NotI sites between the *Renilla* luciferase-coding sequence and the poly(A) site of the psiCHECK-2 plasmid (Promega, Madison, WI, USA) to produce psiC_DAB2. The primers used for the amplification were as follows for DAB2: 5'-TCTAGGCGATCGCTCGAGATTCTGAACTTGGTCTGCAG-3' and 5'-TTATTGCGGCCAGCGGCCGCATTCTGCCACTCCAGTTTATT-3'. Mutations were introduced into the seed sequence of psiC_DAB2 to produce psiC_DAB2m using an EZchange site-directed mutagenesis kit (Enzynomics, Daejeon, South Korea). The primers used for this purpose were as follows: 5'-CGATATTTGGGGTCATGCTAGGCCT-3' and 5'-ACGTAATGTGTTTGGCACAATCACATTTAGC-3'.

### DAB2 over-expression vector

The DAB2 expression vector (pcDNA3.1-DAB2) constructed by Du et al. 30 was used to over-express DAB2 in AGS-EBV cells.

### Luciferase reporter assay

To investigate the effect of miR-BART1-3p upon the expression of DAB2, HEK293T cells or AGS cells seeded in a 96-well plate (5×10^3^ cells/well) were used. After 24 h, the cells were co-transfected with 20 ng psiC_DAB2 and 20 nM miR-BART1-3p or with a seed sequence-mutated miR-BART1-3p (miR-BART1-3pm). Luciferase activity was measured at 48 h post-transfection using a Dual-Glo luciferase reporter assay system (Promega). For each sample, *Renilla* luciferase activity was normalized using firefly luciferase activity.

### Quantitative reverse transcription PCR (qRT-PCR) for DAB2

AGS or AGS-EBV cells were harvested, and the total RNA was extracted using the RNAiso Plus reagent (TaKaRa, Tokyo, Japan) according to the manufacturer's instructions. Next, cDNA was synthesized using 3 μg total RNA, oligo(dT) primer (Macrogen, Seoul, South Korea), and Moloney murine leukemia virus (M-MLV) reverse transcriptase (Invitrogen). Real-time PCR for the indicated genes was carried out using a TOPreal^TM^ Qpcr 2x Pre MIX SYBR-Green kit (Enzynomics, Daejeon, Korea) with the real-time PCR system (CFX96, BioRad, Hercules, CA, USA). The sequences of the primers were as follows: for DAB2, 5'-ATCCTGATCCTTTCCGTGAC-3' and 5'-TCAGCGGAGTAGACGAGCTA-3'; for GAPDH (the glyceraldehyde-3-phosphate dehydrogenase gene), 5'-ATGGGGAAGGTGAAGGTCG-3' and 5'-GGGGTCATTGATGGCAACAATA-3'. PCR conditions were 95 °C for 10 min, followed by 35 cycles at 95 °C for 10 s, 60 °C for 30 s, and 72 °C for 30 s. To confirm the specific amplification of the PCR product, dissociation curves were checked routinely. For this, reaction mixtures were incubated at 95 °C for 60 s and then ramped from 60 to 95 °C at a heating rate of 0.1 °C/s, with fluorescence measured continuously. Relative gene expression was calculated using the quantification cycle (Cq) values, using GAPDH as an internal standard.

### Quantitative reverse transcription PCR for miRNA analysis

The miRNA cDNA was synthesized using a Mir-X miRNA First-Strand synthesis kit (Clontech, Mountain View, CA, USA) according to the manufacturer's instructions. Real-time quantitative PCR procedures were performed using a TOPreal^TM^ Qpcr 2x Pre MIX SYBR-Green kit (Enzynomics, Daejeon, Korea). The forward primer used for miR-BART1-3p amplification was 5'-TAGCACCGCTATCCACTATGTC-3'. All amplifications were performed in triplicate, and Cq values were normalized to the value for an endogenous control, U6, which was supplied in the kit.

### Knocking down of DAB2 expression using small interfering RNA (siRNA)

A small interfering RNA (siRNA) specific for DAB2 (siDAB2) and a control siRNA lacking any known target gene product were synthesized by Genolution Pharmaceuticals (Seoul, South Korea). The sequence of the control siRNA was 5'-CCUCGUGCCGUUCCAUCAGGUAGUU-3'. The sequence of the siDAB was 5'-GGAGUGAGGCCCUAAUGAUUU-3'. AGS-EBV cells (1×10^6^ cells/dish) were transfected with 20 nM siRNA using Lipofectamine 2000 (Invitrogen) in 100-mm-diameter dishes. Cells were harvested to analyze DAB2 expression 48 h after transfection.

### Western blot analysis

Cell lysate in radioimmunoprecipitation assay (RIPA) buffer containing protease inhibitors (1 mM phenylmethylsulfonyl fluoride, 10 μg/ml leupeptin, 10 μg/ml pepstatin A, and 10 μg/ml aprotinin) was mixed with 5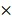 loading buffer (Fermentas, Waltham, MA, USA) and heated at 95 °C for 5 min. Samples were separated electrophoretically on 8% sodium dodecyl sulfate (SDS)-polyacrylamide gels, and the separated proteins were transferred to a polyvinylidene fluoride (PVDF) membrane (Millipore, Billerica, MA, USA). Membranes were blocked and probed with: mouse anti-DAB2 (1:1,000; BD Biosciences, San Jose, CA, USA), or rabbit anti-β-actin (1:3,000; Cell Signaling Technology, Danvers, MA, USA) antibodies. Bound antibodies were detected with horseradish peroxidase (HRP)-conjugated anti-mouse or anti-rabbit secondary antibodies (Santa Cruz, Dallas, TX, USA) at a dilution of 1:5,000 for 45 min at room temperature. Protein bands were visualized using an enhanced chemiluminescence detection system (Amersham Biosciences), and the membrane was exposed to X-ray film (Agfa, Mortsel, Belgium). Anti-β-actin antibody was used to confirm that loading was comparable between gel lanes. The density of each protein band was read and quantified using ImageJ software.

### Propidium iodide (PI) staining

Cells were harvested, washed with phosphate-buffered saline (PBS), and fixed in 70% ethanol at -20 °C overnight. The cells were washed twice with PBS and then resuspended in PBS containing 10 μg/ml RNase A (Invitrogen) and 50 μg/ml PI (Sigma-Aldrich, St. Louis, MO, USA). The distribution of cells in each phase of the cell cycle was analyzed using a FACSCalibur apparatus (BD Biosciences) as described previously 33.

### Annexin V staining

Cells were washed with cold PBS and resuspended in 500 μl of annexin V binding buffer (PE Annexin V Apoptosis Detection kit; BD Biosciences, San Diego, CA, USA), containing phycoerythrin (PE)-labeled annexin V and 7-amino-actinomycin D (7-AAD). Annexin V was used to label cells undergoing apoptosis by detecting phosphatidylserine (PS) on the outer plasma membrane, while 7-AAD was used to detect dead cells. After 10 min of incubation at room temperature in an area shielded from light, the specimens were analyzed by fluorescence-activated cell sorting (FACS) using a FACSCalibur apparatus (BD Biosciences), acquiring 10,000 events. Cells testing positive for annexin V and negative for 7-AAD were considered to be undergoing early apoptosis.

### Wound healing assays

To study the effect of miR-BART1-3p on cell migration, cells (5×10^5^) were seeded into 24-well plates and cultured to reach 90-95% confluence. The cell layer was scratched with a sterile 200-μl pipette tip through the confluent monolayer and washed with PBS to remove cell debris. Four hours later, the cells were transfected with appropriate combinations of siDAB2, LNA-miR-BART1-3p(i), and the DAB2 expression vector. The cells were then cultured in RPMI-1640 medium containing 3% FBS at 37 °C in a humidified chamber with 5% CO_2_. The scratched wounds were observed by Axiovert 200 (Carl Zeiss, Thornwood, NY, USA) microscope just after transfection (time 0) and at 24~48 h after transfection. Photographs were taken to evaluate the level of migration in each group of the transfected cells, and wound areas were assessed by ImageJ software. The experiments were repeated three times.

### Boyden chamber assay

The transfected cells (AGS, AGS-EBV, MKN1, MKN28, SNU-719, or YCCEL1) were plated on 8.0 μm pore size Boyden chambers (Transwell, Corning Life Sciences, Acton, MA, USA) in serum-free medium and 700 μL of medium containing 10% FBS was added to the lower chambers. The chambers were then placed in an incubator at 37˚C with 5% CO_2_ for 24 h. The cells remaining in the upper chamber were then carefully removed. The transwell membrane was fixed with 4% paraformaldehyde for 30 min and stained with 0.1% crystal violet for 10 min. To count the fixed cells, images were captured randomly from 4 fields of vision using inverted microscope (IX70, Olympus, Tokyo, Japan). Three independent experiments were performed to confirm the reproducibility of the results.

### Statistical analyses

The data were analyzed using one-way repeated-measures analysis of variance (ANOVA) or the Student's *t*-test. Curve fitting and analysis were performed using GraphPad Prism software (GraphPad Software, San Diego, CA, USA). P-values less than 0.05 were considered statistically significant. All results were expressed as means ± standard deviations (SDs).

## Results

### DAB2 is down-regulated in EBV-positive gastric carcinoma cells

**Figure 1:**
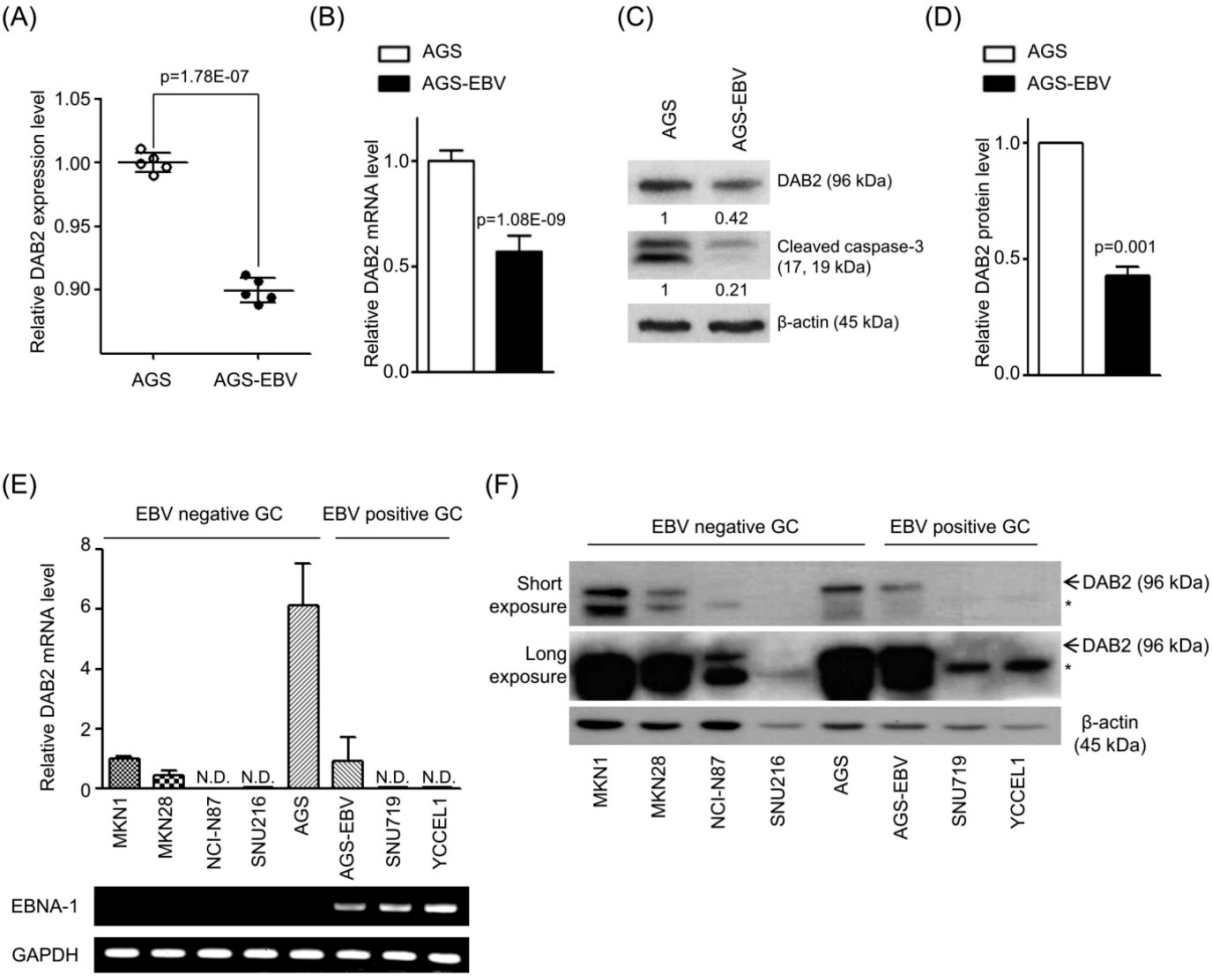
** Reduction of DAB2 expression in EBV-infected gastric cancer cells.** (A) Microarray analysis of AGS and AGS-EBV cells (n=5). (B) DAB2 mRNA expression was measured by real-time RT-PCR using a SYBR Green qPCR kit. (C) DAB2 protein levels were assessed by Western blot analysis using anti-DAB2 (1:1,000) antibody. Anti-β-actin antibody was used to confirm comparable loading. (D) Western blot results similar to those shown in (C) were obtained in two more sets of independently cultured cells. The Western blot results from all three experiments have been normalized to β-actin and are expressed as ratios to the values obtained from AGS cells. Error bars indicate SD (n=3). (E) Real-time RT-PCR analysis of DAB2 mRNA expression was carried out using a SYBR Green qPCR kit. EBNA-1 mRNA was used to confirm EBV infection, as it is known to be expressed in all EBV-infected cells. GAPDH was used as an internal control. (F) DAB2 protein levels were analyzed by Western blot analysis using anti-DAB2 antibody. Anti-β-actin antibody was used to confirm comparable loading. The asterisk (*) marks a non-specific band.

As DAB2 is considered a tumor suppressor in several cancers, we investigated the effect of EBV infection on the expression of DAB2 in gastric carcinoma cells. For this, we analyzed microarray data of AGS and AGS-EBV cells. Notably, DAB2 expression showed a greater reduction in EBV-infected cells (Figure 1A). To validate the array data, DAB2 expression was determined by real-time RT-PCR and Western blot. We found that DAB2 mRNA and protein levels were decreased in AGS-EBV cells by 43% and 58%, respectively, compared to those in AGS cells (Figures 1B-D). These findings indicate that EBV may down-regulate the expression of DAB2 in EBV-associated gastric carcinoma.

We compared the steady-state levels of DAB2 mRNA and protein in EBV-negative (MKN45, NCI-N87, SNU216, AGS) and positive (AGS-EBV, SNU719, YCCEL1) gastric carcinoma cell lines. In general, EBV-negative gastric carcinoma cell lines showed higher DAB2 expression than did EBV-positive gastric carcinoma cell lines (Figures 1E-F). When the films were exposed for a short period of time, a non-specific band was detected for MKN1 and MKN28 cells. These non-specific bands were visible for all of the tested cells when the films were exposed for a longer time period. Curiously, the SNU216 cell line did not express a detectable amount of DAB2, even though it is an EBV-negative GC cell line. The DAB2 gene may be disrupted in this cell line, but we did not investigate this further.

### Screening BART miRNAs that may target DAB2

**Figure 2:**
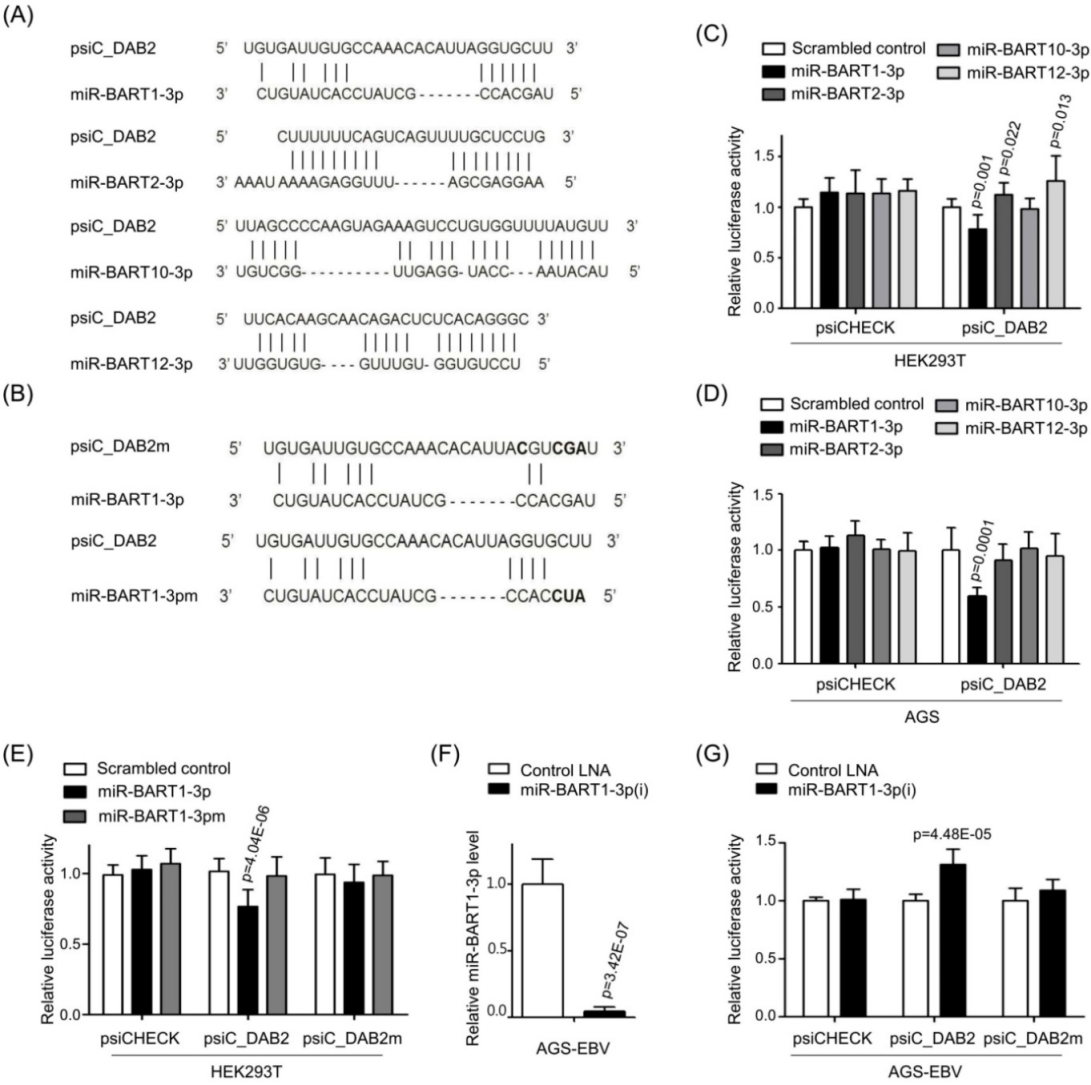
** Screening of BART miRNAs targeting DAB2.** (A) Seed match between BART miRNAs and 3'-UTR of DAB2 mRNA. (B) Seed match between miR-BART1-3p and seed match sequence-mutated (psiC_DAB2m) 3'-UTR of DAB2 mRNA (upper panel), and miR-BART1-3pm and luciferase reporter constructs containing wild type (psiC_DAB2) 3'-UTR of DAB2 mRNA (lower panel). (C-D) Luciferase activity was measured in HEK293T (C) or AGS (D) cells co-transfected with BART miRNA mimics and psiC_DAB2. (E) Luciferase activity was measured in HEK293T cells co-transfected with the miR-BART1-3p mimic or seed sequence-mutated mimic (miR-BART1-3pm) and psiC_DAB2 or psiC_DAB2m. (F) Effect of miR-BART1-3p(i) on the level of miR-BART1-3p. AGS-EBV cells were transfected with 30 nM miR-BART1-3p(i). Real-time RT-PCR analysis of miR-BART1-3p expression was carried out using a SYBR Green qPCR kit. (G) Luciferase activity was measured in AGS-EBV cells co-transfected with the control inhibitor or miR-BART1-3p(i) and psiC_DAB2 or psiC_DAB2m. Luciferase activity was measured 48 h after transfection. A scrambled control and miR-BART1-3pm were used to confirm sequence-specific binding between miR-BART1-3p and the 3'-UTR of DAB2. Luciferase activity was normalized using firefly luciferase activity and expressed as a ratio to the luciferase activity obtained from the scrambled control-transfected cells. Error bars indicate SD (n=3).

To test whether EBV miRNAs down-regulate DAB2 expression in EBV-positive cells, EBV miRNAs showing seed matches with the 3'-UTR of DAB2 were identified using an RNA hybrid program (http://bibiserv.techfak.uni-bielefeld.de/rnahybrid/). Four BART miRNAs (miR-BART1-3p, miR-BART10-3p, miR-BART2-3p, and miR-BART12-3p) were selected to further analyze possible interactions with the DAB2 3'-UTR (Figure 2A).

To investigate whether the BART miRNAs directly target DAB2, we constructed a luciferase reporter vector containing the entire 3'-UTR region of DAB2 (psiC_DAB2). The psiC_DAB2 reporter vector and each of the four BART miRNA mimics were co-transfected into HEK293T or AGS cells. Among the four BART miRNAs tested, only the miR-BART1-3p mimic significantly reduced luciferase activity compared to the scrambled control in both HEK293T cells (Figure 2C) and AGS cells (Figure 2D). The miR-BART2-3p and miR-BART12-3p mimics increased the luciferase activity of psiC_DAB2 (by 1.1-fold and 1.3-fold, respectively) in HEK293T cells (Figure 2C), but not in AGS cells (Figure 2D). The miR-BART10-3p did not inhibit luciferase activity in either of the cell lines. Thus, we selected miR-BART1-3p for further investigation.

### The miR-BART1-3p sequence specifically targets DAB2

To test whether miR-BART1-3p targets the DAB2 sequence specifically, miR-BART1-3pm, which contains a mutation at sites 1-3 of the seed sequence of the miR-BART1-3p (Figure 2B, bottom), was used for a luciferase reporter assay. Unlike miR-BART1-3p, the mutant mimic (miR-BART1-3pm) failed to suppress luciferase activity (Figure 2E). We also substituted sites 2, 3, 4, and 7 of the seed match sequence for miR-BART1-3p in psiC_DAB2 to prepare psiC_DAB2m (Figure 2B, top). When psiC_DAB2m was transfected into HEK293T cells, luciferase activity was not reduced by co-transfected miR-BART1-3p (Figure 2E). These results suggest that miR-BART1-3p inhibits DAB2 expression through a sequence-specific interaction with the 3'-UTR of DAB2 mRNA.

We then tested the effect of endogenously expressed miR-BART1-3p on DAB2 expression in EBV-infected gastric carcinoma cells using an miRNA inhibitor, miR-BART1-3p(i). The miR-BART1-3p(i) suppressed endogenously expressed miR-BART1-3p by over 90% compared to the control LNA in ASG-EBV cells (Figure 2F). When co-transfected into ASG-EBV cells, miR-BART1-3p(i) increased the luciferase activity of psiC_DAB2, but not that of psiC_DAB2m (Figure 2G). These results suggest that the endogenously expressed level of miR-BART1-3p in ASG-EBV was sufficient to suppress DAB2 expression by specifically targeting the 3'-UTR of DAB2.

### miR-BART1-3p regulates DAB2 expression, cell apoptosis, and migration

**Figure 3:**
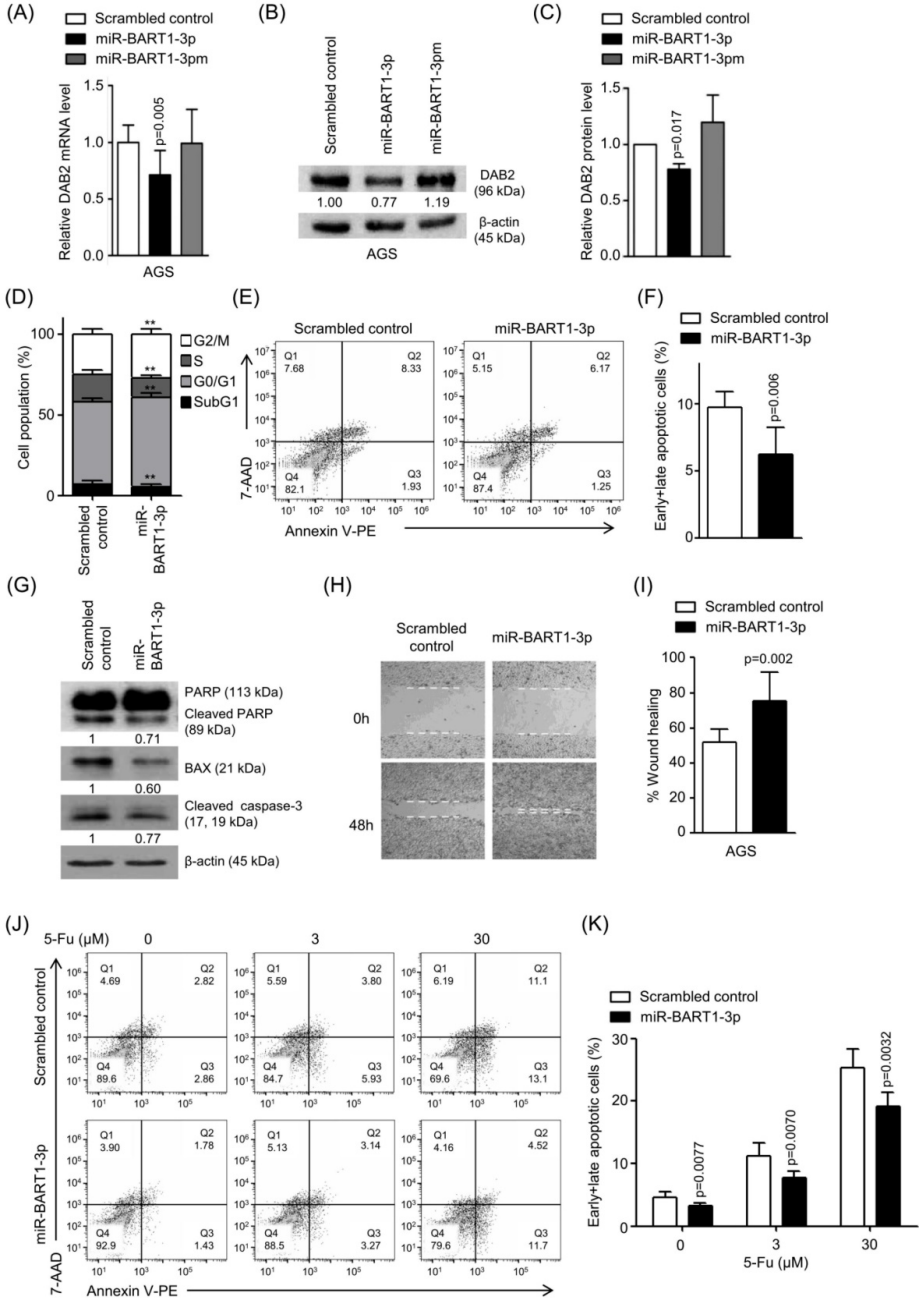
** Effect of miR-BART1-3p on DAB2 expression in AGS cells.** AGS cells were transfected with 30 nM of miR-BART1-3p mimics, miR-BART1-3pm, or the scrambled control. (A) Real-time RT-PCR analysis of DAB2 mRNA expression was carried out using a SYBR Green qPCR kit. (B) DAB2 protein levels were analyzed by Western blot analysis using anti-DAB2 antibody. Anti-β-actin antibody was used to confirm comparable loading. (C) Western blot results similar to those shown in (B) were obtained in two more sets of independently transfected AGS cells. The Western blot results from all three experiments have been normalized to β-actin and are expressed as ratios to the values obtained from the control. (D) Cell cycle analysis was assessed by PI staining 48 h after the cells were transfected with miR-BART1-3p or the scrambled control. The means ± SD values from three independent experiments are plotted (**, p<0.01). (E) Cells were transfected with the miR-BART1-3p mimic or the scrambled control. After 48 h, the proportions of apoptotic cells were evaluated by FACS analysis following PE annexin V staining. (F) Results similar to those in panel (E) were obtained in two more independent experiments, and the mean ± SD values from all three independent experiments are plotted. (G) Apoptosis was assessed by cleaved PARP, BAX, and cleaved caspase-3 protein levels by Western blot analysis. Anti-β-actin antibody was used to confirm comparable loading. (H) Wound healing assays were performed to evaluate the effects of miR-BART1-3p on cell migration in AGS cells. (I) Wound width between the wound edges was evaluated using ImageJ software. The ratios of wound closure compared to the initial wound area from three independent experiments are shown as bar graphs. (J) Cells were transfected with miR-BART1-3p mimic or the scrambled control. Twenty four hours after transfection, the cells were treated with 5-Fu (0, 3, or 30 μM) for 72 h to induce cell apoptosis. The proportions of apoptotic cells were evaluated by FACS analysis following PE annexin V staining. (K) Results similar to shown in panel (J) were obtained in two more independent experiments, and the mean ± SD values from all three experiments are plotted. Error bars indicate SD (n=3).

To confirm the regulatory effect of miR-BART1-3p on DAB2 expression, AGS cells were transfected with miR-BART1-3p, and the mRNA level of DAB2 was analyzed by real-time RT-PCR. When transfected to AGS cells, miR-BART1-3p reduced DAB2 mRNA expression by 27% compared to the scrambled control (Figure 3A). Likewise, Western blot analysis revealed approximately 23% reduced DAB2 protein levels following miR-BART1-3p transfection (Figures 3B-C). As expected, miR-BART1-3pm failed to affect the levels of DAB2 mRNA and protein expression (Figures 3A-C). These results support that the miR-BART1-3p sequence specifically down-regulates DAB2 expression.

We then investigated the biological function of miR-BART1-3p on cell death and migration ability. AGS cells were transfected with the miR-BART1-3p mimic or the scrambled control. After 48 h, apoptotic cells were measured by FACS analysis following PI or PE annexin V staining assay (Figures 3D-F). To further investigate the effect of miR-BART1-3p on apoptosis, we analyzed apoptosis following PI staining. AGS cells transfected with miR-BART1-3p showed decreased sub-G1 cell populations (5.4 ± 1.8%) compared to the scrambled control-transfected AGS cells (6.9 ± 2.2%). miR-BART1-3p transfection increased the proportions of cells in the G0/G1 and G2/M phases, while it decreased the proportions of cells in the S stage compared to scrambled control transfection (Figure 3D). Similarly, PE annexin V staining showed that apoptotic cell population (9.7 ± 1.2%) was decreased in the miR-BART1-3p transfected AGS cells compared to the scrambled control-transfected cells (6.2 ± 2.0%) (Figures 3E-F). Furthermore, cleaved caspase-3, cleaved PARP, and BAX protein levels were significantly decreased in the miR-BART1-3p transfected AGS cells compared to the control cells (Figure 3G). In addition, the capability of miR-BART1-3p to reduce apoptosis induced by 5-Fu in AGS cells was also analyzed. For this, cells were transfected with miR-BART1-3p 24 h prior to the 5-Fu treatment. The cells were harvested 72 h after transfection, and stained with PE annexin V. FACS analysis showed that miR-BART1-3p efficiently suppressed cell apoptosis induced by 5-Fu (Figure 3J-K).

We next investigated whether miR-BART1-3p could reduce DAB2 expression in some other EBV negative GC cell lines, MKN1, and MKN28 (Figure 1F). In these cells, miR-BART1-3p reduced DAB2 mRNA expression compared to the scrambled control (Figure S1A). Western blot analysis revealed that DAB2 protein levels were also decreased following miR-BART1-3p transfection than following the scrambled control transfection (Figures S1B-C).

The effect of miR-BART1-3p on 5-Fu induced cell apoptosis of MKN1 and MKN28 cells was also assessed. When the cells were transfected with miR-BART1-3p, cleaved caspase-3, and cleaved PARP protein levels were significantly decreased than when the cells were transfected with the scrambled control (Figure S1D).

As several studies have reported a role of DAB2 in cell migration 34, we examined the effect of miR-BART1-3p on cell migration. Wound healing assays showed that AGS cell migration was significantly enhanced following miR-BART1-3p transfection as compared to that following the scrambled control transfection (Figures 3H-I). In addition, the Boyden chamber assay showed that miR-BART1-3p increased migration of AGS, MKN1, and MKN28 cells compared to the scrambled control (Figure S1E-F).

### The EBV miR-BART1-3p inhibitor blocks the effect of miR-BART1-3p for cell apoptosis and migration in EBV-infected GC cells

**Figure 4:**
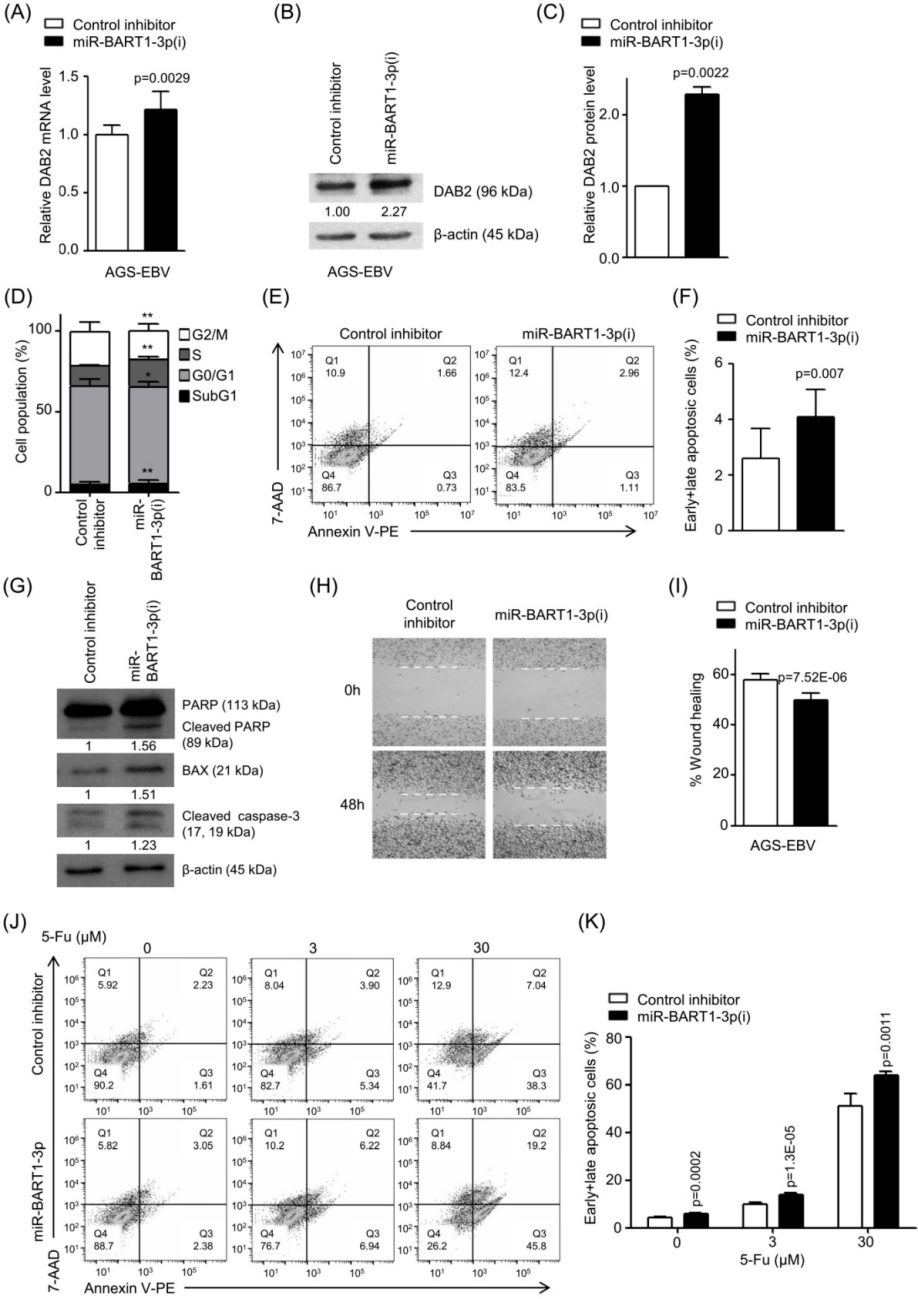
** Effect of miR-BART1-3p(i) in AGS-EBV cells.** AGS-EBV cells were transfected with 30 nM miR-BART1-3p(i) or the control inhibitor. (A) Real-time RT-PCR analysis of DAB2 mRNA expression was carried out using a SYBR Green qPCR kit. (B) DAB2 protein levels were analyzed by Western blot analysis using anti-DAB2 antibody. Anti-β-actin antibody was used to confirm comparable loading. (C) Western blot results similar to those shown in (B) were obtained in two more sets of independently transfected AGS-EBV cells. The Western blot results have been normalized to β-actin and are expressed as ratios to the values obtained from the control. (D) Cell cycle analysis was assessed by PI staining 48 h after the cells were transfected with miR-BART1-3p(i) or the control inhibitor. The means ± SD values from three independent experiments are plotted (*, p<0.05; **, p<0.01). (E) Cells were transfected with miR-BART1-3p(i) or the control inhibitor. After 48 h, the proportions of apoptotic cells were assessed by PE annexin V staining. (F) Results similar to those in panel (E) were obtained in two more independent experiments, and the means ± SD from all three independent experiments are plotted. (G) Apoptosis was assessed by cleaved PARP, BAX, and cleaved caspase-3 protein levels by Western blot analysis. Anti-β-actin antibody was used to confirm comparable loading. (H) A wound healing assay was performed to evaluate the effects of miR-BART1-3p(i) on cell migration in AGS-EBV cells. (I) Wound width between the wound edges was evaluated using ImageJ software. The relative ratios of wound closure compared to the initial wound area from three independent experiments are shown as bar graphs. (J) Cells were transfected with miR-BART1-3p(i) or the control inhibitor. Twenty four hours after transfection, the cells were treated with 5-Fu (0, 3, or 30 μM) for 72 h to induce cell apoptosis. The proportions of apoptotic cells were evaluated by FACS analysis following PE annexin V staining. (K) Results similar to shown in panel (J) were obtained in two more independent experiments, and the mean ± SD values from all three experiments are plotted. Error bars indicate SD (n=3).

We further investigated whether an endogenously expressed level of miR-BART1-3p was sufficient to affect DAB2 expression in EBV-infected GC cells. Real-time RT-PCR and Western blot were performed to assess DAB2 levels after transfection with miR-BART1-3p(i) and the control inhibitor. In AGS-EBV cells, the DAB2 mRNA level was increased by 22% (Figure 4A), and DAB2 protein levels were enhanced by 2.2-fold following miR-BART1-3p(i) transfection compared to that following the control inhibitor transfection (Figures 4B-C). These results indicate that endogenously expressed miR-BART1-3p efficiently inhibits DAB2 expression in AGS-EBV cells.

To analyze the effect of inhibited miR-BART1-3p on cell apoptosis and migration, AGS-EBV cells were transfected with miR-BART1-3p(i) or the control inhibitor. FACS analysis following PI staining revealed that the sub-G1 population was increased (5.4 ± 2.0%) in miR-BART1-3p(i) transfected AGS-EBV cells compared to the control inhibitor-transfected cells (4.6 ± 2.0%).

miR-BART1-3p(i) transfection decreased the proportions of cells in both the G0/G1 and G2/M phases, while it increased the proportions of cells in the S phase compared to control inhibitor transfection (Figure 4D). PE annexin V staining showed that miR-BART1-3p(i) transfection increased the apoptotic cell ratio (4.1 ± 1.0%) more than the control inhibitor transfection did in AGS-EBV cells (2.6 ± 1.1%) (Figures 4E-F). Accordingly, the expression levels of cleaved PARP, BAX, and cleaved caspase-3 proteins were significantly increased in miR-BART1-3p(i) transfected AGS-EBV cells compared to those in the control cells (Figure 4G). In addition, PE annexin V staining showed that apoptosis induced by 5-Fu further increased following transfection with miR-BART1-3p(i) (Figure 4J-K).

As all the tested EBV positive cell lines expressed little DAB2, we investigated endogenous miR-BART1-3p level in these cells. Naturally EBV-infected GC cell lines, SNU-719, and YCCEL1, showed higher miR-BART1-3p expression level than artificially EBV-infected AGS-EBV cells (Figure S2A).

To investigate the role of miR-BART1-3p in EBV-positive GC cell lines, we transfected SNU-719 and YCCEL1 cells with miR-BART1-3p(i). Endogenously expressed miR-BART1-3p was dramatically suppressed by miR-BART1-3p(i) (Figure S2B). We then tested the effect of miR-BART1-3p(i) on 5-Fu induced apoptosis of these cells. Western blot results showed that cleaved caspase-3 and cleaved PARP protein levels were significantly increased in the miR-BART1-3p(i)-transfected SNU-719 and YCCEL1 cells compared to those in the control cells (Figure S2C).

Wound healing was suppressed in miR-BART1-3p(i) transfected AGS-EBV cells compared to that in the control inhibitor-transfected cells (Figures 4H-I). Likewise, the Boyden chamber assay also revealed that miR-BART1-3p(i) hindered migration of AGS-EBV, SNU-719, and YCCEL1 cells (Figure S2D-E).

### Knockdown of DAB2 using siRNA reduces cell apoptosis and induces cell migration in AGS and AGS-EBV cells

**Figure 5:**
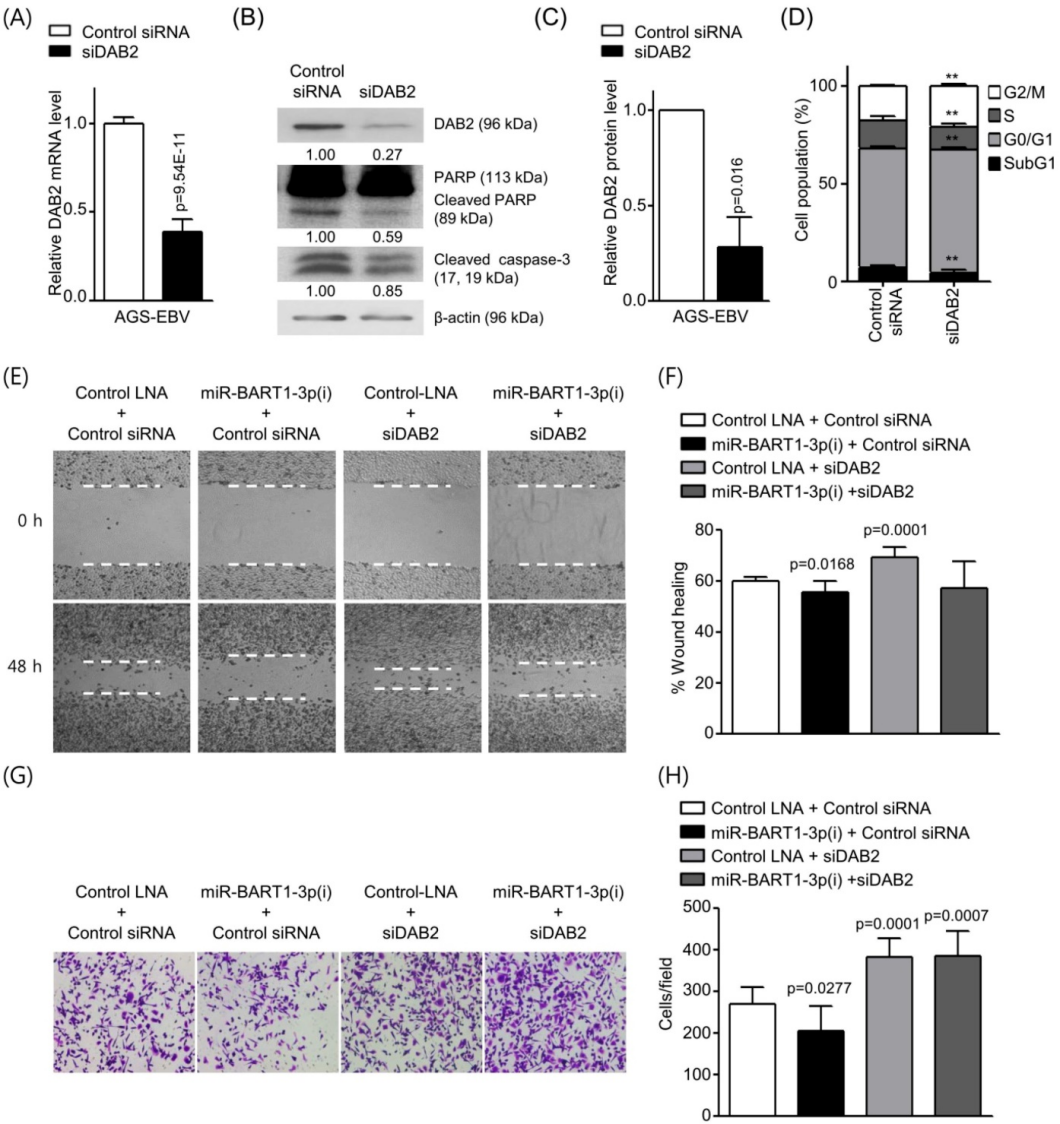
** Effect of DAB2 knockdown using siDAB2 in AGS-EBV cells.** AGS-EBV cells were transfected with 20 nM siDAB2 or a control siRNA. (A) Real-time RT-PCR analysis of DAB2 mRNA expression was carried out using a SYBR Green qPCR kit. (B) DAB2, PARP, cleaved PARP, and cleaved caspase-3 protein levels were analyzed by Western blot analysis. Anti-β-actin antibody was used to confirm comparable loading. (C) DAB2 protein levels as shown in (B) were obtained in two more sets of independently transfected AGS-EBV cells. The Western blot results have been normalized to β-actin and are expressed as ratios to the values obtained from the control. (D) Cell cycle analysis was assessed by PI staining 48 h after the cells were transfected with siDAB2 or the control siRNA. The means ± SD values from three independent experiments are plotted (**, p<0.01). (E) A wound healing assay was performed to evaluate the effects of miR-BART1-3p(i) and siDAB2 on cell migration in AGS-EBV cells. (F) Wound width between the wound edges was evaluated using ImageJ software. The relative ratios of wound closure compared to the initial wound area from three independent experiments are shown as bar graphs. (G) Boyden chamber assays were performed to evaluate the effects of miR-BART1-3p(i) and siDAB2 on cell migration in AGS-EBV cells. (H) Results similar to shown in panel (G) were obtained in two more independent experiments, and the mean ± SD values from all three experiments are plotted. Error bars indicate SD (n=3).

To investigate whether siRNA against DAB2 (siDAB2) also leads to a phenotype similar to that achieved with miR-BART1-3p, siDAB2 was transfected into AGS-EBV cells. After 48 h, real-time RT-PCR and Western blot analysis were performed to assess DAB2 expression levels. The siDAB2 transfection of AGS-EBV cells efficiently knocked down DAB2 mRNA (approximately 61%) and protein (approximately 73%) expression (Figures 5A-C). As reduction of DAB2 by miR-BART1-3p inhibited apoptosis, we inferred that DAB2 might regulate cell apoptosis. To test this possibility, we explored the role of siDAB2 in controlling cell apoptosis. The proportion of apoptotic cells was reduced in AGS-EBV cells following transfection with siDAB2 (4.3 ± 1.6%) than with the control siRNA (7.1 ± 1.2%). siDAB2 transfection increased the proportions of cells in both the G0/G1 and G2/M phases, while it decreased the proportions of cells in the S phase compared to control siRNA transfected cells (Figure 5D). Furthermore, the levels of cleaved caspase-3 and PARP were decreased more in siDAB2-transfected AGS-EBV cells than in the control cells (Figure 5B). These indicate that DAB2 causes apoptosis of AGS-EBV cells.

Wound healing and Boyden chamber assays showed that cell migration was decreased with up-regulation of DAB2 by miR-BART1-3p(i), while migration was increased by siDAB2 compared to the control group. The effect of miR-BART1-3p(i) on cell migration was abrogated by co-transfected siDAB2 (Figures 5E-H). These findings indicate that miR-BART1-3p inhibits apoptosis and promotes cell migration by directly inhibiting DAB2 in AGS-EBV cells.

We next inhibited DAB2 expression in AGS cells using siDAB2 to investigate whether knockdown of DAB2 shows similar effects as miR-BART1-3p. The siDAB2 efficiently knocked down DAB2 protein expression (approximately 77%) in AGS cells (Figure S3A). DAB2 knockdown using siDAB2 decreased the levels of cleaved caspase-3 and cleaved PARP more than in the control cells (Figure S3B). The proportion of apoptotic AGS cells was reduced following transfection with siDAB2 (2.4 ± 0.9%) compared to the control siRNA (3.8 ± 0.5%). siDAB2 transfection increased the proportions of cells in both the G0/G1 and G2/M phases, while it decreased the proportion of cells in the S phase compared to control siRNA transfection (Figure S3C). Furthermore, the wound healing and Boyden chamber assays showed that cell migration was increased following siDAB2 transfection (Figure S3D-G).

### DAB2 over-expression promotes cell apoptosis and inhibits cell migration in AGS-EBV cells

**Figure 6:**
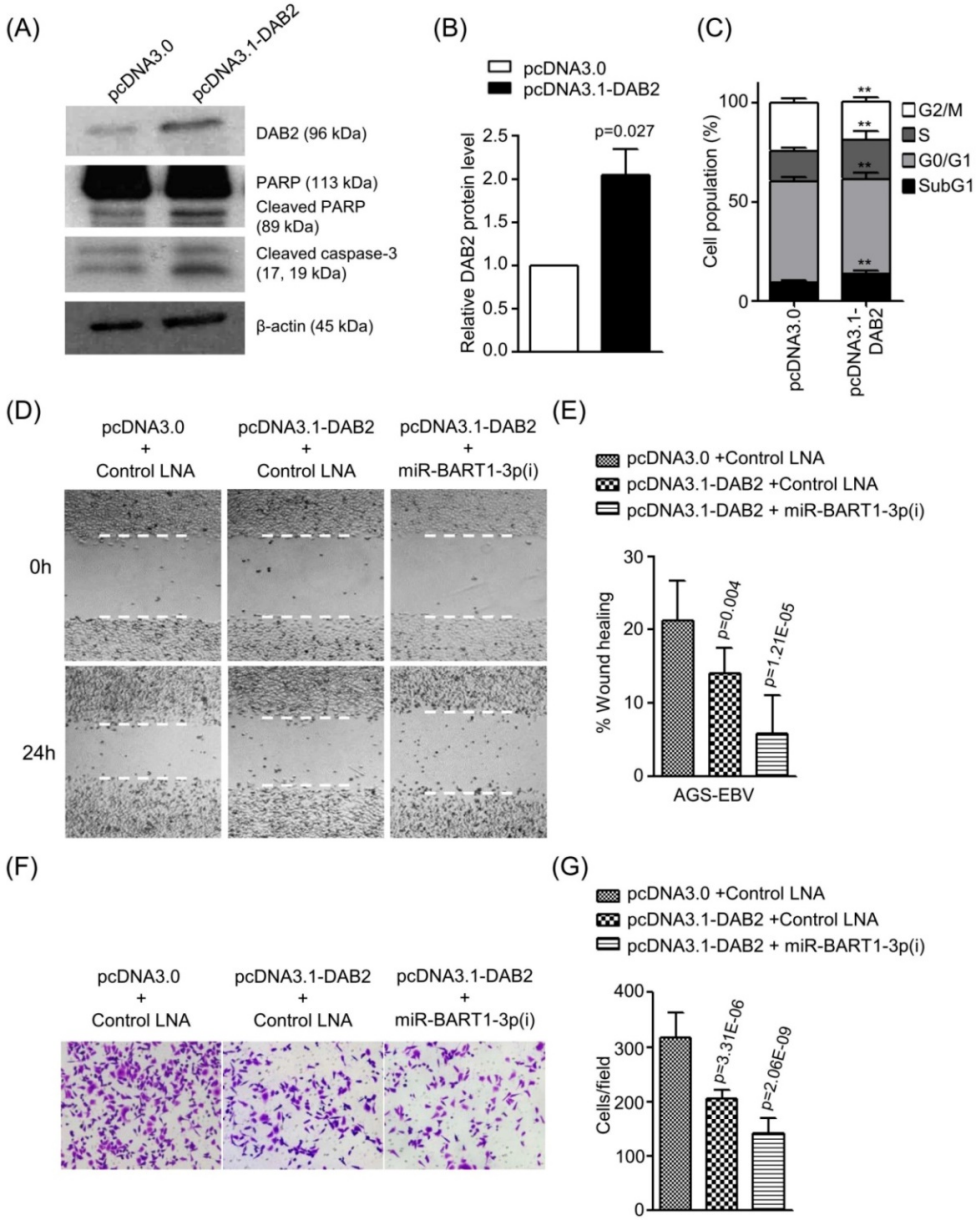
** Effect of DAB2 over-expression in AGS-EBV cells.** AGS-EBV cells were transfected with the DAB2 expression vector (pcDNA3.1-DAB2) or an empty vector (pcDNA3.0). (A) DAB2, PARP, cleaved PARP, and cleaved caspase-3 protein levels were analyzed by Western blot. Anti-β-actin antibody was used to confirm comparable loading. (B) DAB2 protein levels as shown in (A) were obtained in two more sets of independently transfected AGS-EBV cells. All three Western blot results have been normalized to the level of β-actin and are expressed as ratios to the values obtained from the control. (C) Cell cycle analysis was assessed by PI staining 48 h after the cells were transfected with pcDNA3.1-DAB2 or pcDNA3.0. The means ± SD values from three independent experiments are plotted (**, p<0.01). (D) A wound healing assay was performed to evaluate the effects of pcDNA3.1-DAB2 and miR-BART1-3p(i) on cell migration in AGS-EBV cells. (E) Wound width between the wound edges was evaluated using ImageJ software. The relative ratios of wound closure compared to the initial wound area from three independent experiments are shown as bar graphs. (F) Boyden chamber assays were performed to evaluate the effects of DAB2 overexpression and miR-BART1-3p(i) transfection on cell migration in AGS-EBV cells. (G) Results similar to shown in panel (F) were obtained in two more independent experiments, and the mean ± SD values from all three experiments are plotted. Error bars indicate SD (n=3).

To confirm the role of DAB2 in EBV-positive gastric carcinoma cells, we tested the effect of DAB2 over-expression using pcDNA3.1-DAB2 on apoptosis and cell migration in AGS-EBV cells. Western blotting was performed to evaluate DAB2 levels after transfection of pcDNA3.1-DAB2 or a control vector (pcDNA3.0). The DAB2 protein level was increased by 2-fold following pcDNA3.1-DAB2 transfection than following the control vector transfection (Figures 6A-B). The transient over-expression of DAB2 in AGS-EBV cells significantly increased apoptotic cells (Figures 6A and 6C) and decreased cell migration ability (Figures 6D-G). When AGS-EBV cells were co-transfected with the pcDNA3.1-DAB2 vector and miR-BART1-3p(i), migration was further enhanced than when cells were transfected with each of them alone (Figures 6D-E). The Boyden chamber assay also showed similar results (Figure 6F-G). These results may be attributable to the inhibitory effects of miR-BART1-3p on cell apoptosis and DAB2 expression, and demonstrate that the inhibitory function of DAB2 on cell migration is regulated by miR-BART1-3p.

## Discussion

We observed low DAB2 expression in EBV-infected gastric cancer cell lines compared with EBV-negative GC cell lines. An EBV miRNA miR-BART1-3p suppressed the expression of DAB2 by specifically and directly targeting its 3'-UTR, while miR-BART1-3p(i) suppressed DAB2 expression. Transfection of miR-BART1-3p suppressed cell apoptosis and promoted cell migration. As expected, siDAB2 showed similar effects with miR-BART1-3p on apoptosis and migration. In contrast, increasing DAB2 expression by miR-BART1-3p(i) or pcDNA3.1-DAB2 transfection resulted in opposite effects on cell migration and apoptosis.

Our results showing lower DAB2 expression in EBV-positive GC cell lines than in EBV-negative cells support that DAB2 functions as a tumor suppressor in EBVaGC. That is consistent with previous reports showing DAB2 as a putative tumor suppressor in various cancer types 21-31. Some EBV-negative GC cells which do not express miR-BART1-3p showed no or very little expression of DAB2. Here we did not investigate the mechanisms that down-regulated DAB2 in those cells. Perhaps other mechanisms such as promoter methylation or cellular miRNAs in addition to miR-BART1-3p, might play roles in suppressing DAB2 expression. Further studies are required to deeply understand the regulation of DAB2 in these cells.

Chen et el. 35 demonstrated that most of the highly abundant EBV miRNAs, including miR-BART1-3p, share their seed sequences with human miRNAs. Furthermore, they found that those human miRNAs were dysregulated in human malignancies, suggesting a potential linkage between highly coordinated mechanisms through which EBV miRNAs could mimic or compete with human miRNAs to affect cellular functions 35. The miR-29 family shares a seed sequence with miR-BART1-3p. While most studies have suggested a tumor suppressor function of miR-29 with down-regulated expression in multiple human carcinoma including GC, some reports showed oncogenic functions of the miR-29 family 36-38. Whether dysregulation of the miR-29 family sharing a seed sequence with miR-BART1-3p can be responsible for DAB2 regulation, and whether coordinated action between miR-BART1-3p and miR-29 affects viral life cycle, are questions that warrant further investigation.

We observed that miR-BART1-3p plays an important regulatory role in the apoptosis and migration of EBVaGC. Other groups have also reported oncogenic contributions of miR-BART1-3p in EBV-associated carcinomas. Cai et al. 39 demonstrated that EBV-miR-BART1 (both miR-BART1-3p and miR-BART1-5p) was highly expressed in NPC and that it increased the migration and invasion of NPC cells by directly targeting the cellular tumor suppressor PTEN. Shinozaki-Ushiku et al. 40 demonstrated that the expression level of miR-BART1-3p was highest among all of the EBV miRNAs in epithelial tumors. These findings together with our data suggest that miR-BART1-3p may play a critical role for tumorigenesis in EBVaGC.

In general, miRNA-mediated gene suppression is thought to regulate about 30% of target genes' expression 41. However, a much larger reduction in DAB2 expression was observed in naturally EBV-infected GC cells compared to EBV-negative cells. Other mechanisms in addition to miR-BART1-3p-mediated suppression may also contribute to suppression of DAB2 expression in EBVaGC. DAB2 promoter hypermethylation is known to be common in human malignant cancers, and there are multiple CpG sites in the promoter of DAB2 21, 26, 42, 43. EBVaGC cases show a higher prevalence of DNA hypermethylation 44. Thus, in addition to the BART miRNA-mediated post-transcriptional regulation, DAB2 promoter hypermethylation may also contribute to low DAB2 expression in EBVaGC.

Previous observation shows that adaptor protein DAB2 is involved in multiple pathway signaling through binding motifs (c-Src 19, Wnt 20, and TGF-β 45, 46). Wong AM et al. 47 reported that significantly up-regulated EBV miRNAs may target pathway signaling such as Wnt, MAPK, and TGF-β, which are closely associated with cell cycle arrest, apoptosis, and migration. Our data suggests that the miR-BART1-3p/DAB2 axis regulates cell cycle, migration, and apoptosis presumably by modulating various signaling pathways involving DAB2 in EBV-associated GC. More delicate mechanistic studies are warranted to uncover the detailed mechanism.

In conclusion, our data suggest that EBV suppressed cell apoptosis and promoted cell migration in GC cells by miR-BART1-3p-mediated DAB2 targeting. Our findings support that DAB2 may have a tumor-suppressive function in EBVaGC.

## Supplementary Material

Supplementary figures.
