# Supplementary material for: Epstein-Barr virus miR-BART1-3p suppresses apoptosis and promotes migration of gastric carcinoma cells by targeting DAB2
Source: Int J Biol Sci. 2020 Jan 14;16(4):694–707. doi: 10.7150/ijbs.36595 (PMC6990914; doi:10.7150/ijbs.36595)

Figure legends

Supplementary Figure 1. Effect of miR-BART1-3p on DAB2 expression in MKN1 and MKN28. MKN1 or MKN28 cells were transfected with 30 nM of miR-BART1-3p mimics or the scrambled control. (A) Real-time RT-PCR analysis of DAB2 mRNA expression was carried out using a SYBR Green qPCR kit. (B) DAB2 protein levels were analyzed by Western blot analysis using anti-DAB2 antibody. Anti-β-actin antibody was used to confirm comparable loading. The asterisk (*) marks a non-specific band. (C) Western blot results similar to those shown in (B) were obtained in two more sets of independently transfected MKN1 or MKN28 cells. The Western blot results from all three experiments have been normalized to β-actin and are expressed as ratios to the values obtained from the control. (D) Apoptosis was assessed by detecting cleaved PARP and cleaved caspase-3 protein levels by Western blot analysis in the cells transfected with miR-BART1-3p. Twenty four hours after transfection, the cells were treated with 30 μM of 5-Fu for 72 h to induce cell apoptosis. Anti-β-actin antibody was used to confirm comparable loading. (E) Boyden chamber assays were performed to evaluate the effects of miR-BART1-3p on cell migration in AGS, MKN1, or MKN28 cells. (F) Results similar to shown in panel (E) were obtained in two more independent experiments, and the mean ± SD values from all three experiments are plotted. Error bars indicate SD (n=3).

Supplementary Figure 2. Effect of miR-BART1-3p(i) in SNU-719 and YCCEL1. (A) Comparison of miR-BART1-3p levels in EBV positive GC cell lines. (B) Effect of miR-BART1-3p(i) on the level of miR-BART1-3p. SNU-719 or YCCEL1 cells were transfected with 30 nM miR-BART1-3p(i). Real-time RT-PCR analysis of miR-BART1-3p expression was carried out using an SYBR Green qPCR kit. (C) Apoptosis was assessed by measuring cleaved PARP and cleaved caspase-3 protein levels by Western blot analysis in the cells transfected with miR-BART1-3p(i). Twenty four hours after transfection, the cells were treated with 10 μM of 5-Fu for 72 h to induce cell apoptosis. Anti-β-actin antibody was used to confirm comparable loading. (D) Boyden chamber assays were performed to evaluate the effects of miR-BART1-3p(i) on cell migration in AGS-EBV, SNU-719, or YCCEL1 cells. (E) Results similar to those shown in panel (D) were obtained in two more independent experiments, and the mean ± SD values from all three experiments are plotted. Error bars indicate SD (n=3).

Supplementary Figure 3. Effect of DAB2 knockdown using siDAB2 in AGS cells. AGS cells were transfected with 20 nM siDAB2 or a control siRNA. (A) DAB2 protein levels were analyzed by Western blot analysis using anti-DAB2 antibody. Anti-β-actin antibody was used to confirm comparable loading (top). The Western blot results from three independent experiments were normalized to β-actin and are expressed as ratios to the values obtained from the control (bottom). (B) Apoptosis was assessed by measuring cleaved PARP and cleaved caspase-3 protein levels by Western blot analysis in AGS cells transfected with siDAB2. Twenty four hours after transfection, the cells were treated with 30 μM of 5-Fu for 72 h to induce cell apoptosis. Anti-β-actin antibody was used to confirm comparable loading. (C) Cell cycle analysis was assessed by PI staining 48 h after the cells were transfected with siDAB2 or the control siRNA. The means ± SD values from three independent experiments are plotted (**, $p<0.01$). (D) A wound healing assay was performed to evaluate the effects of siDAB2 on cell migration in AGS cells. (E) Wound width between the wound edges was evaluated using ImageJ software. The relative ratios of wound closure compared to the initial wound area from three independent experiments are shown as bar graphs. (F) Boyden chamber assays were performed to check the effect of siDAB2 on cell migration in AGS cells. (G) Results similar to shown in panel (F) were obtained in two more independent experiments, and the mean ± SD values from all three experiments are plotted. Error bars indicate SD (n=3).

Supplementary Figure 1.

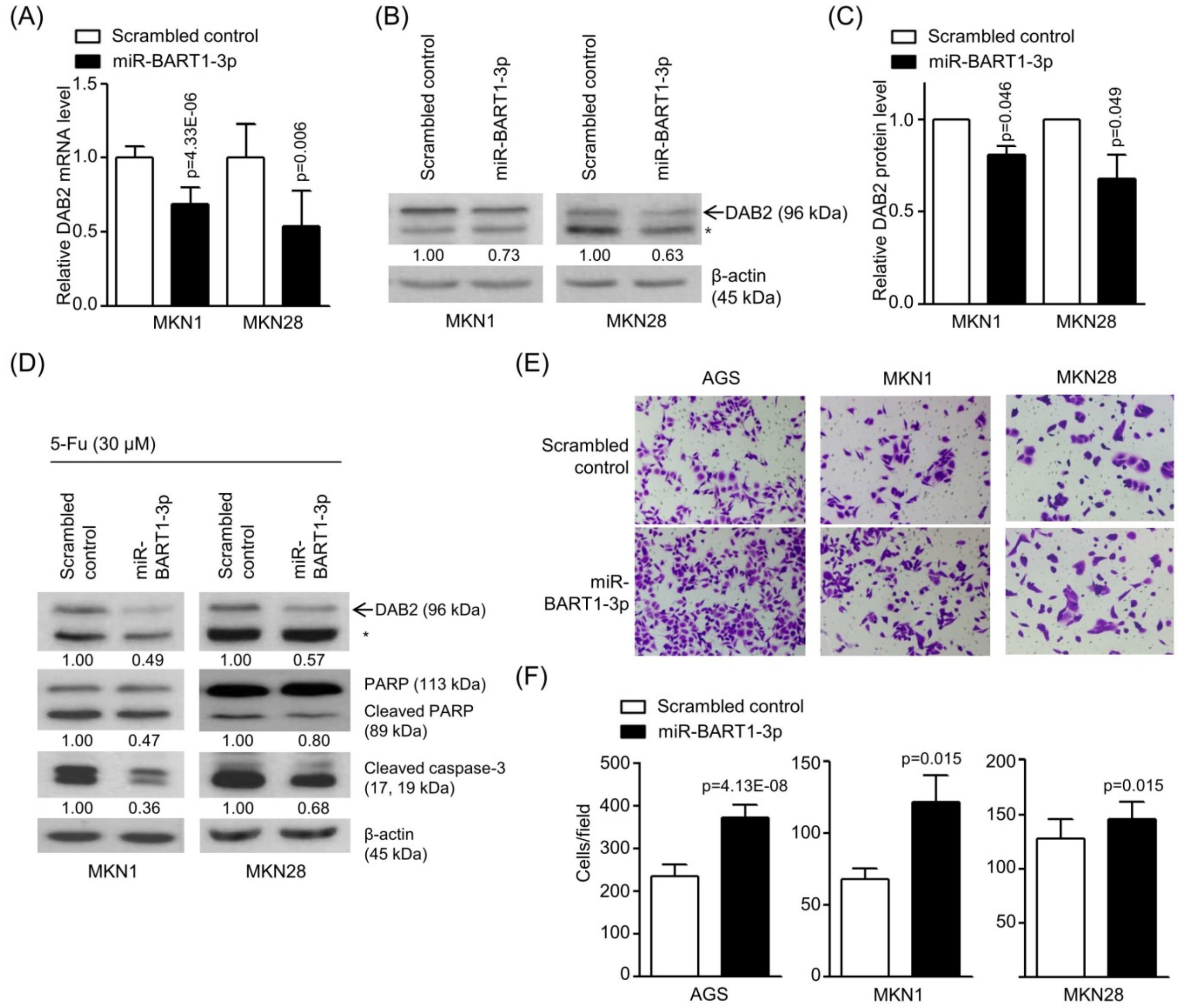

Supplementary Figure 2.

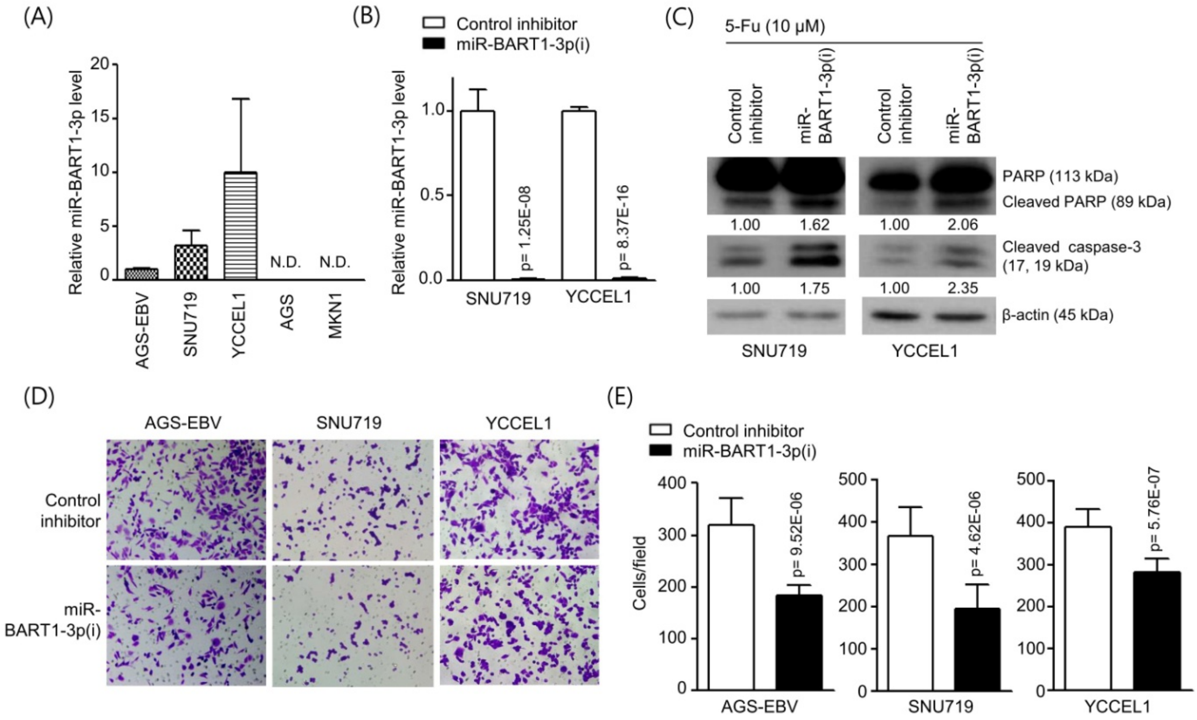

Supplementary Figure 3.

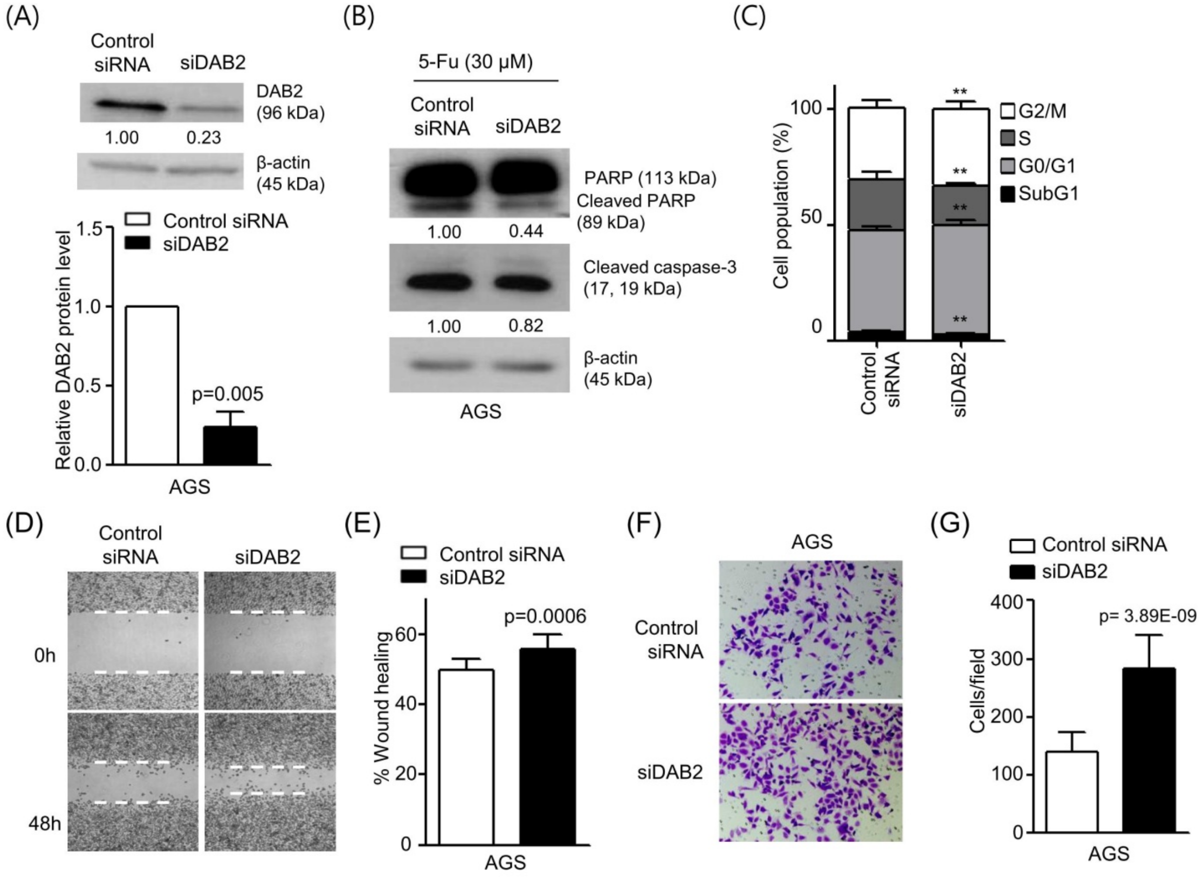

Supplement: Supplementary file 1 — Supplementary figures. [file ijbsv16p0694s1.pdf]
